# Anxiety-Induced Sleep Disturbance and Associated Lifestyle Behaviors According to Sex in Argentine Adolescents

**DOI:** 10.3389/fnbeh.2022.860241

**Published:** 2022-04-25

**Authors:** José Francisco López-Gil, Iván Cavero-Redondo, Pedro J. Tárraga López, Estela Jiménez-López, Alberto Durán González, Irene Sequí-Domínguez, Arthur Eumann Mesas

**Affiliations:** ^1^Health and Social Research Center, Universidad de Castilla-La Mancha, Cuenca, Spain; ^2^Facultad de Ciencias de la Salud, Universidad Autónoma de Chile, Talca, Chile; ^3^Department of Medical Sciences, Faculty of Medicine, Universidad de Castilla-La Mancha, Albacete, Spain; ^4^Department of Psychiatry, Hospital Virgen de La Luz, Centro de Investigación Biomédica en Red de Salud Mental (CIBERSAM), Cuenca, Spain; ^5^Postgraduate Program in Public Health, Universidade Estadual de Londrina, Londrina, Brazil

**Keywords:** physical activity, screen time, eating healthy, adolescence, youths

## Abstract

**Purpose:**

The aim of the current study was twofold: first, to determine the prevalence of anxiety-induced sleep disturbances among Argentine adolescents according to sex, and second, to identify the association between these sleep disturbances and lifestyle behaviors in this population.

**Methods:**

This is a cross-sectional study with data from the Global School-based Student Health Survey (GSHS) in Argentina (2018). A total of 32,393 adolescents (aged 12–17 years; 53.4% girls) were included in the final analysis. Anxiety-induced sleep disturbances were assessed with the question “During the past 12 months, how often have you been so worried about something that you could not sleep at night?”

**Results:**

The prevalence of anxiety-induced sleep disturbances was higher in girls (17.4%) than in boys (7.9%) (*p* < 0.001). In boys, results indicated that those who used marijuana (cannabis) (odds ratio [OR] = 1.46, 95% confidence interval [CI] 1.08–1.98), used amphetamine or methamphetamine (OR = 2.19, 95% CI 1.28–3.77), walked or biked to or from school (OR = 1.53, 95% CI 1.19–1.96), and spent 3 h or more in sedentary behaviors (OR = 1.35, 95% CI 1.05–1.74) were more likely to report anxiety-induced sleep disturbances. In girls, those who ate from a fast-food restaurant (OR = 1.24, 95% CI 1.05–1.47), consumed alcoholic beverages (OR = 1.45, 95% CI 1.19–1.75), smoked cigarettes (OR = 2.09, 95%CI 1.05–4.14), consumed any tobacco product (OR = 1.47, 95% CI 1.19–1.82), used amphetamine or methamphetamine (OR = 2.08, 95% CI 1.33–3.26), and those who spent 3 h or more in sedentary behaviors (OR = 1.32, 95% CI 1.11–1.57) were more likely to report frequent anxiety-induced sleep disturbances.

**Conclusion:**

In conclusion, considerable sex differences were observed with respect to the prevalence of anxiety-related sleep disturbances and associated lifestyle aspects.

## Introduction

Anxiety is a leading cause of sleep disturbance in adolescents (Mullin and Simon, 2017), while sleep disturbance is associated with an increased risk of developing symptoms of anxiety (Armstrong et al., 2014; Mullin and Simon, 2017). Available data indicate that sleep disturbances are quite common in children with anxiety disorders (de Zambotti et al., 2018; Haugland et al., 2021). For instance, a recent study among Norwegian adolescents found that total anxiety symptoms were associated with several sleep variables (e.g., insomnia, short sleep duration, long sleep onset latency) among adolescents with and without a risk for depression, after adjusting for age and sex (Haugland et al., 2021). Both sleep problems and anxiety are commonly associated with other psychiatric disorders and are independent risk factors for substance use, cardiovascular diseases, and suicide in adolescence, suggesting that treatment of both in early adolescence may reduce the risk for adverse outcomes (de Zambotti et al., 2018). Difficulties falling asleep and awakenings during nights are often caused by anxiety disorders, and these disorders (anxiety and sleep) are bi-directionally associated, suggesting that each contributes to the development and is a consequence of one another (Alvaro et al., 2013). In this sense, Vancampfort et al. (2018) showed that the overall prevalence of anxiety-induced sleep disturbance, defined as being worried about something that keeps you awake at night, was 7.8% in their study involving 181,093 adolescents from 67 countries. Other authors have used a more comprehensive definition of anxiety-induced sleep disturbance, considering it as a condition of low sleep quality, lack of sleep or clinical insomnia as a consequence of loneliness (either social or emotional), worries, sleep disturbance, and disproportionate fear (Joyce-Beaulieu and Sulkowski, 2016; Ahinkorah et al., 2021b).

Adolescence is a major stage of risk for the development of anxiety symptoms and syndromes that can range from mild transient symptoms to complete anxiety disorders (Beesdo et al., 2009). Furthermore, adolescence is an age phase in which sleep is of critical importance (Owens and Weiss, 2017). Adequate sleep is another key factor in promoting healthy development in the young population, and the risk of health problems, such as mental disorders, is increased as a consequence of sleep deprivation and unstable sleep routines (Owens and Weiss, 2017; Schneider et al., 2018). Strikingly, although cognitive-behavioral treatments seem to be effective for anxiety and sleep disturbances in adolescents (Kendall and Peterman, 2015; de Zambotti et al., 2018), few prevention strategies are available (Cabral and Patel, 2020). From a public health perspective, knowing the potential risk factors associated with anxiety-induced sleep disturbances is crucial to developing interventions to prevent sleep disturbances in adolescents (Beesdo et al., 2009).

On the other hand, adolescence is also a critical phase for sex differences in sleep alterations. Whereas the prevalence of sleep problems is comparable between girls and boys during childhood (Fricke-Oerkermann et al., 2007; Zhang et al., 2009), these tend to be more common in girls during this age phase (Blank et al., 2015; Zhang et al., 2016). Additionally, some sex differences have been found regarding factors associated with sleep disturbances (Zhang et al., 2016).

Furthermore, lifestyle behaviors have been shown to affect sleep. For instance, Phillips et al. (1975) found in their experiment that dietary habits (e.g., type of foods and timing of consumption) influence sleep duration. On the other hand, a systematic review with meta-analysis performed by Córdova et al. (2018) showed an inverse cross-sectional relationship between sleep duration in children and unhealthy dietary habits, especially with higher consumption of energy-dense food (e.g., snacks, soda) and lower consumption of fruits and vegetables. In adolescents, a cross-sectional study performed in Italy showed that participants with shorter sleep duration were more likely to eat fewer vegetables and fruits and to eat away more often (Ferranti et al., 2016).

In addition, the relationship between sleep problems and movement-related behaviors has been studied. In a meta-analysis of cross-sectional studies, increased physical activity (PA) and reduced sedentary behavior were associated with greater psychosocial health in young people (Rodriguez-Ayllon et al., 2019). In youth, a systematic review and meta-analysis conducted by Lang et al. (2016) showed that adolescents with increased PA are more likely to have adequate sleep according to objective and subjective measures of both PA and sleep. Similarly, prolonged sedentary behavior has been cross-sectionally related to greater anxiety-induced sleep disturbances in the existing literature (Vancampfort et al., 2018; Werneck et al., 2020).

Similarly, the consumption of psychoactive substances such as alcohol, tobacco, and illicit drugs (e.g., marijuana, amphetamines, etc.) has been reported to have a harmful influence on sleep quality among adolescents (Bartel et al., 2015). Indeed, the consumption of some substances has been associated with poor sleep duration and increased nocturnal arousal (Kotagal and Pianosi, 2006). Likewise, one study conducted among US adolescents showed an association of sleep problems with substance use or smoking (Zhang et al., 2017). In summary, substance use disorders can cause or aggravate sleep problems, which in turn can lead to addictions to these and other psychoactive substances, thus closing the vicious circle (Reid-Varley et al., 2020).

To date, the role of lifestyle factors associated with anxiety-induced sleep disturbance among adolescents has been little explored. Understanding this relationship could be helpful for adolescents to adopt lifestyle changes to reduce anxiety-induced sleep disturbance, which could result in improved mental health. Considering that both anxiety disorders and sleep problems at young ages increased considerably during the COVID-19 pandemic (Wang et al., 2022), the relationship between the two conditions has gained increased attention and requires the expansion of the available body of scientific evidence. In addition, considering the sex differences found in both the prevalence of sleep disturbance (Blank et al., 2015; Zhang et al., 2016) and its associated factors (Zhang et al., 2016), it seems of relevance to know the specific lifestyle factors differences between sexes, in order to promote more appropriate prevention and intervention programs.

Therefore, this study examined data from a nationally representative sample of Argentine adolescents to address two objectives: first, to determine the prevalence of anxiety-induced sleep disturbances and, second, to identify the association between these sleep disturbances and healthy lifestyle behaviors in this population. These objectives have been analyzed separately for boys and girls because the progression of pubertal maturation varies according to sex and may generate, for example, differences in the prevalence of insomnia symptoms (Zhang et al., 2016).

## Materials and Methods

### Design and Sample

This is a cross-sectional study using data from the Global School-based Student Health Survey (GSHS) in Argentina (2018). The GSHS is a school-based survey that applies self-administered questionnaires to acquire information on people’s health behavior and protective factors associated with the leading causes of morbidity and mortality worldwide.

A two-stage cluster sampling strategy was designed to obtain representative data for all students in eight first-grade primary/polymodal schools and 12 fifth-grade polymodal schools in Argentina. First, schools with probability proportional to entry size were chosen. Second, classes were randomly selected, and all students in these classes were eligible to participate. Further information on the specific methodological methods used in the GSHS can be obtained from the World Health Organization (WHO) website^1^ and the Centers for Disease Control and Prevention (CDC) website.^2^ A total of 56,981 students participated in the Argentina GSHS. Of these students, 21,201 (37.2%) were excluded because of missing body mass index data. Additionally, 3,387 (5.9%) were excluded due to missing data for other variables of interest (i.e., self-reported sleep problems, physical activity, and substance use). Finally, a total of 32,393 (56.8%) students were included in the final analysis.

### Procedures

#### Anxiety-Induced Sleep Disturbance

For anxiety-induced sleep disturbances (dependent variable), the following question was used: “During the past 12 months, how often have you been so worried about something that you could not sleep at night?” The response options were 1-never, 2-rarely, 3-sometimes, 4-most of the time, and 5-always. For further analysis, this variable was dichotomized into no anxiety-induced sleep disturbance (“never,” “rarely,” and “sometimes”) and anxiety-induced sleep disturbance (“most of time” and “always”). In the absence of a specific cut-off point to determine anxiety-induced sleep disturbance, this choice was based on the categorization made in previous studies (Vancampfort et al., 2018; Smith et al., 2020; Werneck et al., 2020; Ahinkorah et al., 2021a,b).

#### Lifestyle Behaviors

Table 1 describes the results of the measurement of lifestyle behaviors (independent variables) in the present study. Lifestyle behaviors were categorized into three groups: eating behaviors (fruit, vegetable, soft drink, and fast-food intake), movement-related behaviors (physical activity, sedentary behavior, and active commuting), and substance use behaviors (alcohol, cigarettes, any tobacco product, marijuana, and amphetamines/methamphetamines).

**TABLE 1:** Global School-based Health Survey questions included in the analysis of lifestyle correlates related to anxiety-induced sleep disturbance.

Variable	Question	Codification
**Eating behavior**		
Fruit intake	During the past 7 days, how many times did you eat fruit?	0 = 0–1 time per day 1 = 2 or more times per day
Vegetable intake	During the past 7 days, how many times did you eat vegetables?	0 = 0–1 time per day 1 = 2 or more times per day
Soft drink intake	During the past 7 days, how many times did you drink a can, bottle, or glass of a carbonated soft drink?	0 = 0 times per day 1 = At least one time per day
Fast-food intake	During the past 7 days, on how many days did you eat food from a fast-food restaurant?	0 = 0 days per week 1 = At least 1 day per week
**Movement behavior**		
Physically active	During the past 7 days, on how many days were you physically active for a total of at least 60 min per day?	0 = 0–6 days per week 1 = 7 days per week
Sedentary behavior	How much time do you spend during a typical or usual day sitting and watching television, playing computer games, talking with friends, or doing other seated activities such as surfing the Internet?	0 = Less than 3 h per day 1 = 3 or more hours per day
Active commuting	During the last 7 days, on how many days did you walk or ride a bicycle to or from school?	0 = 0 days per week 1 = At least one time per week
**Substance use behavior**		
Alcohol consumption	During the past 30 days, on how many days did you have at least one drink containing alcohol?	0 = 0 days per month 1 = At least 1 day per month
Smoke cigarettes	During the past 30 days, on how many days did you smoke cigarettes?	0 = 0 days per month 1 = At least 1 day per month
Any tobacco products use	During the past 30 days, on how many days did you use any tobacco products other than cigarettes?	0 = 0 days per month 1 = At least 1 day per month
Marijuana use	During the past 30 days, how many times have you used marijuana?	0 = 0 times per month 1 = At least one time per month
Amphetamines and methamphetamines use	During your life, how many times have you used amphetamines or methamphetamines?	0 = 0 times per month 1 = At least one time per month

#### Potential Covariates

Sex and age were self-reported. Height and weight were assessed with the following questions: “How tall are you without your shoes on?” and “How much do you weigh without your shoes on?” respectively. Body mass index (BMI) was computed by dividing the weight (kg)/height (m^2^) of the participants. BMI z-scores were determined following the World Health Organization (WHO) criteria (Onis et al., 2007). Perceived hunger (used as a proxy of socioeconomic status) was determined through the following question: “During the past 30 days, how often did you go hungry because there was not enough food in your home?” Responses varied from 1-never, 2-rarely, 3-sometimes, 4-most of the time, to 5-always. These covariates were selected according to scientific evidence on their potential confounding effect on the associations of interest (Rethorst et al., 2014; Vancampfort et al., 2018; Ahinkorah et al., 2021a).

### Statistical Analysis

Data are shown as the mean and standard deviation for continuous variables and numbers and frequencies for categorical variables. Differences between continuous and categorical variables were examined by Student’s *t*-test and the chi-square test, respectively. An exploratory analysis was conducted to define the percentage of adolescents reporting anxiety-induced sleep disturbance. In preliminary analyses, a statistically significant interaction between all analyzed lifestyle factors and sex in relation to anxiety-induced sleep disturbances (*p* < 0.05 for all) was observed. Consequently, all the analyses were stratified by sex. Backward logistic regression analyses were performed to investigate the association between lifestyle factors (independent variable) and anxiety-induced sleep disturbances (dependent variable). First, binary logistic regression analyses were performed including all the lifestyle variables. Second, the lifestyle variable with the highest *p*-value was excluded from the model for each step of the backward logistic regression. Thus, exclusion of lifestyle variables from the model was stopped once all lifestyle variables had a *p* < 0.10. Third, variables included in the last step with *p* < 0.05 were considered significantly associated with anxiety-induced sleep disturbance. All analyses were adjusted by age, BMI z-score, and perceived hunger (as a proxy of socioeconomic status). We used the survey functions in STATA 16.1 (StataCorp, College Station, TX, United States) to perform all analyses and account for weighting for each observation. Statistical significance was represented by a *p*-value < 0.05.

### Ethics

All GSHS surveys have been previously approved in each country by the corresponding national government agency and by an institutional ethics committee or board. The protocol was approved by an independent ethics committee in Argentina and by the Pan American Health Organization’s Ethics Review Committee. Survey administration was authorized by the Ministry of Education, provincial ministries, and school authorities. Responses were completely confidential and anonymous in unnamed answer sheets, which were collected at the end of class. Students were required to provide their verbal consent before completing the survey.

## Results

Table 2 shows the characteristics of the adolescents included in this study by sex. The mean ± standard deviation of age was slightly higher in boys (15.1 ± 1.3) than in girls (15.0 ± 1.3) (*p* = 0.012). The prevalence of excess weight (overweight/obesity) was higher in boys (43.3%) than in girls (31.2%) (*p* < 0.001). Finally, the prevalence of anxiety-induced sleep disturbances was higher in girls (17.4%) than in boys (7.9%) (*p* < 0.001).

**TABLE 2:** Characteristics of the study participants according to sex (*N* = 32,393).

Variables	Boys (*n* = 15,095)	Girls (*n* = 17,298)	
	M (SD)/n (%)	n (%)	*p*
Age (years)	15.1 (1.3)	15.0 (1.3)	0.012
**Age group**			
12	30 (0.2)	26 (0.2)	0.003
13	2,292 (15.2)	2,855 (16.5)	
14	3,260 (21.6)	3,601 (20.8)	
15	3,345 (22.2)	3,974 (23.0)	
16	3,569 (23.6)	3,987 (23.0)	
17	2,599 (17.2)	2,855 (16.5)	
Height (m)	1.69 (0.10)	1.60 (0.07)	<0.001
Weight (kg)	64.1 (15.3)	56.8 (13.3)	<0.001
BMI (z-score)	0.62 (1.19)	0.45 (1.07)	<0.001
Overweight/Obesity^a^	6,543 (43.3)	5,404 (31.2)	<0.001
**Perceived hungry**			
Never	10,567 (70.0)	11,849 (68.5)	<0.001
**Anxiety-induced sleep disturbance**			
Never	5,469 (36.4)	3,357 (19.5)	<0.001
Rarely	4,833 (32.1)	5,121 (29.7)	
Sometimes	3,547 (23.6)	5,746 (29.2)	
Most of the time	890 (5.9)	2,227 (12.9)	
Always	306 (2.0)	778 (4.5)	
Most of the time/Always^b^	1,196 (7.9)	3,005 (17.4)	<0.001

*^a^Prevalence of excess of weight according to the World Health Organization criteria (Onis et al., 2007). ^b^Anxiety-induced sleep disturbance defined as the sum of adolescents who reported that they could not sleep “most of the time” and “always.” BMI, Body mass index.*

**FIGURE 1:**
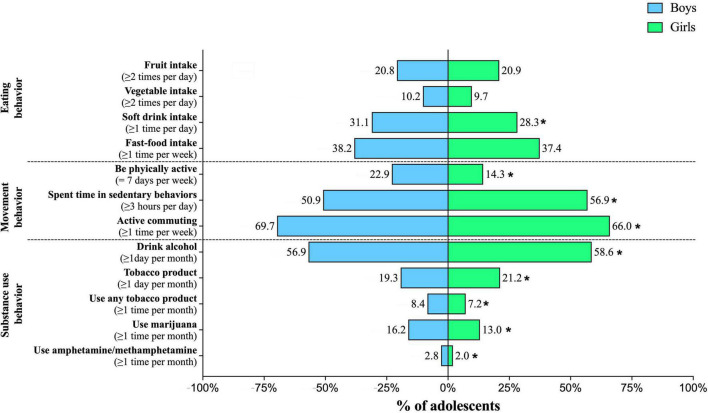
Prevalence of different lifestyle behaviors stratified by sex.

Figure 1 shows the prevalence of different lifestyle behaviors stratified by sex. Regarding movement-related behaviors, boys showed a higher prevalence of active commuting and being physically active (*p* < 0.001 for all), as well as less time spent in sedentary behaviors (*p* < 0.001). According to substance use behavior, the prevalence of consumption of alcohol and cigarettes was higher in girls (*p* < 0.001 for all). Conversely, the prevalence of amphetamine/methamphetamine use, marijuana (cannabis), and any tobacco product use were higher in boys than in girls (*p* < 0.001 for all). In the case of eating behavior, significant differences were only found for soft drink intake (*p* < 0.001), with a higher prevalence for boys.

Finally, Table 3 depicts the lifestyle behaviors retained in the last step of the multivariate model. The backward logistic regression analysis included seven steps for boys and four for girls, in which the least statistically significant correlation was removed from the model at each step. In boys, the results indicated that those who consumed marijuana (cannabis) (odds ratio [OR] = 1.46, 95% confidence interval [CI] 1.08–1.98), consumed amphetamine or methamphetamine (OR = 2.19, 95% CI 1.28–3.77), walked or biked to or from school (OR = 1.53, 95% CI 1.19–1.96), and spent 3 h or more in sedentary behaviors (OR = 1.35, 95% CI 1.05–1.74) were more likely to report anxiety-induced sleep disturbance. In girls, those who ate at a fast-food restaurant (OR = 1.24, 95% CI 1.05–1.47), consumed alcoholic beverages (OR = 1.45, 95% CI 1.19–1.75), smoked cigarettes (OR = 2.09, 95% CI 1.05–4.14), consumed any tobacco products (OR = 1.47, 95% CI 1.19–1.82), consumed amphetamine or methamphetamine (OR = 2.08, 95% CI 1.33–3.26), and those who spent 3 h or more in sedentary behaviors (OR = 1.32, 95% CI 1.11–1.57) were more likely to report frequent anxiety-induced sleep disturbances.

**TABLE 3:** Final result of the univariate multilevel logistic regression analysis with anxiety-induced sleep disturbance as a dependent variable and potential lifestyle correlates as independent variables, among Argentine adolescents by sex.

Predictors	Boys (*n* = 14,516)			Girls (*n* = 16,872)		
	OR	95% CI	*p*	OR	95% CI	*p*
**Eating habits**						
Fast-food intake						
≥1 day per week	Variable excluded in step 4^a^			1.24	(1.05–1.47)	0.014
0 days per week				1		
**Movement**						
Active commuting						
≥1 time per week	1.53	(1.19–1.96)	0.001	Variable excluded in step 2^a^		
0 times per week	1					
Sedentary behavior						
≥3 or more hours per day	1.35	(1.05–1.74)	0.019	1.32	(1.11–1.57)	0.001
<3 h per day	1			1		
**Drugs**						
Alcohol consumption						
≥1 day per month	Variable excluded in step 2^a^			1.45	(1.19–1.75)	<0.001
0 days per month				1		
Smoke cigarettes						
≥1 day per month	Variable excluded in step 3^a^			1.47	(1.19–1.82)	<0.001
0 days per month				1		
Any tobacco product use						
≥1 day per month	Variable excluded in step 1^a^			1.59	(1.17–2.16)	0.003
0 days per month				1		
Marijuana use						
≥1 time per month	1.46	(1.08–1.98)	0.014	Variable excluded in step 1^a^		
0 times per month	1					
Amphetamine or methamphetamine use						
≥1 time per month	2.19	(1.28–3.77)	0.004	2.08	(1.33–3.26)	0.001
0 times per month	1			1		

*Data indicated as odds ratio and 95% confident intervals. Adjusted by age, body mass index (z-score), and perceived hunger.*

*^a^Logistic regression models with backward stepwise variable selection method (statistical criterion p > 0.20).*

## Discussion

The current study aimed to evaluate the prevalence of anxiety-induced sleep disturbance and lifestyle behaviors linked to this condition in a nationally representative sample of Argentine adolescents. A marked sex difference was found in this cross-sectional study with respect to the prevalence of anxiety-related sleep disturbances, which was more than twice as high in girls as in boys. This finding agrees with previous studies that found that anxiety-related sleep disturbances are more prevalent among girls (Pengpid and Peltzer, 2020, 2022; Werneck et al., 2020). Regarding the causes of inadequate sleep in adolescents, internal biological processes have been identified, such as the normal change (delay) in circadian rhythm that occurs in relation to puberty and a development-based slowing of the “sleep drive” (Owens and Weiss, 2017). Thus, sex differences in sleep problems (e.g., anxiety-induced sleep disturbances) during adolescence are especially recognized, due to biological, behavioral, or social factors. Regarding biological factors, sexual maturation has been suggested as a factor that could influence sleep problems, since it is influenced by the changing the hormone milieu (Marsella and Sharkey, 2020). Similarly, the menstrual cycle could influence sleep, due to variations in sex steroid hormones (Baker et al., 2020). In relation to social factors, it has been suggested that girls may be more likely to have anxiety-induced sleep disturbances because they experience adverse life events (e.g., domestic violence) more frequently than boys, which could exacerbate the development of anxiety disorders (Biaggi et al., 2016). Supporting this notion, exposure to violence (e.g., intentional injury, physical attack, physical fight) has been positively associated with higher odds of anxiety-induced insomnia among adolescents (Smith et al., 2020). Finally, the coexistence of specific anxiety disorders with substance use in adolescence (especially in girls) has also been identified as a relevant social factor (Wu et al., 2010).

Considerable sex differences were also observed with respect to lifestyle aspects associated with anxiety-related sleep disturbance. According to eating behavior, anxiety-induced sleep disturbance was associated with eating fast food from a restaurant at least once (or more) per week in girls. In this regard, one study showed that adolescents who had a high intake of fast food were more than three times more likely to report anxiety-induced sleep disturbance (Khan et al., 2017). Similarly, the consumption of ultra-processed foods, such as those available in fast food restaurants, has been associated with elevated anxiety-induced sleep disturbance among Brazilian adolescents (Werneck et al., 2020). The absence of a significant association in boys could be explained because this eating behavior possibly has less influence on sleep problems compared to others more frequent in boys than in girls, such as active commuting and marijuana use. There are some reasons that might explain the association between anxiety-induced sleep disturbances and fast food. First, sleep problems could affect dietary choices, which have previously been associated with shorter sleep duration and might play a crucial role in mediating the relationship between sleep and adolescent health (Kruger et al., 2014). Second, fast food often contains high glycemic index carbohydrates. In this regard, some authors have advised that hyperglycemia following a high glycemic index diet and subsequent compensatory hyperinsulinemia may stimulate the release of autonomic counter-regulatory hormones (e.g., cortisol, adrenaline, growth hormone, glucagon), which could exert an impact on sleep disturbances (Gais et al., 2003; Gangwisch et al., 2020). Similarly, foods with a high glycemic index have been shown to enhance inflammatory immune responses (Kim et al., 2018) and cause alterations in the gut microbiome, which could also deeply influence sleep quality (Gérard and Vidal, 2019). Third, fast food also tends to contain more saturated fatty acids. Supporting this notion, a higher intake of saturated fats during the day has been linked to a shorter duration of slow-wave sleep (Grandner et al., 2010; St-Onge et al., 2016) and more arousal episodes at night (St-Onge et al., 2016). However, caution is required to interpret these results, since studies on the role of saturated fatty acids on sleep are relatively scarce (Zhao et al., 2020).

Furthermore, we found associations between anxiety-induced sleep disturbances and movement-related behaviors. Thus, our results showed that spending three or more hours in sedentary behaviors was likely related to anxiety-induced sleep disturbances in both sexes. In this regard, Vancampfort et al. (2018) provided strong multinational evidence on the relationship between anxiety-induced sleep disturbances and sedentary behaviors in adolescents. Possible reasons for these findings are that some sedentary behaviors, such as excessive media use and prolonged overnight exposure, may influence circadian rhythms and relocate sleep timing (Primack et al., 2009; Soundy et al., 2014). In addition, the literature has also suggested that inactivity could alter serum melatonin levels and lead to changes in nocturnal melatonin release (Vancampfort et al., 2018). Additionally, exposure to blue spectrum light through screens may also influence this relationship (LeBourgeois et al., 2017). Conversely, although physical activity has been found in the scientific literature to be associated with longer sleep durations (Lang et al., 2016), in this study, the association between physically active conditions and sleep disturbances was not statistically significant in either sex. This lack of association could be justified by the use of subjective measures applied to determine the level of physical activity in the GSHS surveys. Furthermore, these questions only ask about the number of days above 60 min of moderate-to-vigorous physical activity, an aspect that has been questioned in the new guidelines on physical activity in children and adolescents (Bull et al., 2020), which suggests that the counting of minutes of physical activity should be considered globally per week and not by isolated days.

In relation to substance use behavior, we found an association between some substances and anxiety-induced sleep disturbance in both sexes. An association with higher illicit substance use was found in girls (e.g., alcohol, cigarettes, any tobacco product) than in boys (e.g., marijuana), which is consistent with a previous study among US adolescents (Wu et al., 2010). However, the relatively lower prevalence of anxiety-induced sleep disturbance in boys than in girls could (at least partially) explain the lack of statistical significance. Adolescents are generally more vulnerable to tobacco, alcohol, and cannabis than adults (Spear, 2016). Our results are in agreement with the findings of Sivertsen et al. (2015) in their study of Norwegian adolescents, who found that all measured sleep indicators were associated with substance involvement. Similarly, one longitudinal study showed that trajectories of sleep health were linked to trajectories of cannabis and alcohol use during late adolescence and early adulthood (Troxel et al., 2021). Additionally, one study of adolescents from sub-Saharan Africa found that cannabis use was related to anxiety-induced sleep disturbances (Ahinkorah et al., 2021a). Regarding amphetamine or methamphetamine use, in adults, D-amphetamine caused a marked decrease in sleep drive and harmful effects on certain elements of recovery sleep (Waters et al., 2003). Likewise, amphetamine-type stimulants are the most potent agents that promote wakefulness by blocking dopamine reuptake and stimulating dopamine release (Wisor et al., 2001). Moreover, in another study it was observed that e-cigarette (as tobacco-related products) users were more predisposed to have shorter sleep durations than non-users of e-cigarette (Wiener et al., 2020). There are other reasons that could explain these results. First, many e-cigarettes have stimulants (e.g., nicotine) that are known to influence sleep (Wiener et al., 2020). For instance, nicotine influences the sleep-wake cycle through nicotine receptors, promoting wake time and decreasing total sleep time, as well as rapid eye movement sleep (Siqueira and Committee on Substance Use and Prevention, 2017). Second, concomitant changes in circadian rhythms and the reward function of the brain that is activated when engaging in risky behaviors could be an influential factor (Hasler et al., 2014). Furthermore, human circadian genetic variability is closely related to substance use (Blomeyer et al., 2013), and this association is likely to be bidirectional: adolescents with addiction problems often have altered circadian rhythms, and certain chronotypes have also been shown to increase the risk of substance abuse (Logan et al., 2014). Supporting this idea, circadian disruption and sleep problems have been associated with higher alcohol consumption (Hasler et al., 2015).

This study is not without limitations. First, because of the cross-sectional design, cause-and-effect relationships cannot be established from the present findings. Second, although previous studies have assessed anxiety-induced sleep disturbance by the same single-item question (Vancampfort et al., 2018; Werneck et al., 2020; Ahinkorah et al., 2021a), we cannot adequately determine the perseverance and specific type of anxiety-induced sleep disturbance reported by adolescents. This item did not provide in-depth data outcome data. However, it is complex to use more specific methodologies regarding psychological outcomes in national epidemiological studies. Future studies using validated questionnaires are required to acquire more detailed information about this outcome. Third, this study included self-reported information from adolescents, which may result in some bias. For instance, this fact may be especially relevant for the illicit substance use. However, to reduce this bias, information from GHSH did not include school or student identifiers in the public use data set (i.e., anonymous data). Furthermore, in this study height and weight were self-reported by adolescents which, in addition to the large loss of participants due to missing values or outliers, could also have introduced an information bias in the present findings (Gorber et al., 2007). Fourth, information about race/ethnicity was not assessed, which could influence the results obtained. Finally, it is important to note that the classification of behaviors and outcomes into only two categories, although it is a common procedure in epidemiological studies and facilitates comparison of the results with those of other authors, limits the possibility of analyzing the effect of different levels of adherence to certain lifestyle habits. In contrast, the present study has some strengths. For instance, this study included a large nationally representative sample of Argentine adolescents. This fact allowed us to obtain a high statistical power and to analyze the association between a large number of lifestyle variables and anxiety-induced sleep disturbances, stratified by sex. In this sense, although previous studies adjusted their analyses by sex (Haugland et al., 2021), our findings indicate that the study of factors associated with anxiety-induced sleep disturbances in adolescents requires separate analyses for boys and girls due to biological and behavioral differences between the sexes. Additionally, this study adds information to the scientific knowledge about the understudied relationship between lifestyle behaviors and anxiety-induced sleep disturbances among adolescents.

## Conclusion

Our results indicate a high prevalence of anxiety-related sleep disturbances, specifically among girls, and point to sex-specific lifestyle issues related to these disorders. Lifestyle behaviors, such as eating habits, movement-related behavior, and substance use, are associated with the prevalence of anxiety-induced sleep disturbances, which could affect adolescent health. Programs and strategies to encourage a healthy lifestyle throughout adolescence and that take into account sex specificity are necessary and should focus on actions considering the importance of the noted lifestyle behaviors in decreasing the prevalence of anxiety-induced sleep disturbances.

## Data Availability Statement

Publicly available datasets were analyzed in this study. This data can be found here: https://extranet.who.int/ncdsmicrodata/index.php/catalog/866.

## Ethics Statement

All GSHS surveys have been previously approved in each country by the corresponding national government agency and by an institutional ethics committee or board. The protocol was approved by an independent ethics committee in Argentina and by the Pan American Health Organization’s Ethics Review Committee. Survey administration was authorized by the Ministry of Education, provincial ministries, and school authorities. Responses were completely confidential and anonymous in unnamed answer sheets, which were collected at the end of class. Students were required to provide their verbal consent before completing the survey. Written informed consent to participate in this study was provided by the participants’ legal guardian/next of kin.

## Author Contributions

JL-G: conceptualization, methodology, software, validation, analysis, data curation, and writing—original draft preparation. AM and IC-R: supervision. AM, PT, EJ-L, AG, IS-D, and IC-R: writing—review and editing. All authors have read and agreed to the published version of the manuscript.

## Conflict of Interest

The authors declare that the research was conducted in the absence of any commercial or financial relationships that could be construed as a potential conflict of interest.

## Publisher’s Note

All claims expressed in this article are solely those of the authors and do not necessarily represent those of their affiliated organizations, or those of the publisher, the editors and the reviewers. Any product that may be evaluated in this article, or claim that may be made by its manufacturer, is not guaranteed or endorsed by the publisher.

## Footnotes

1
http://www.who.int/ncds/surveillance/gshs/en/

2
https://www.cdc.gov/gshs/index.htm
